# Chrononutrition and cardiometabolic health: circadian timing as a dimension of precision nutrition

**DOI:** 10.3389/fnut.2026.1779033

**Published:** 2026-06-03

**Authors:** Wafa Alotaibi

**Affiliations:** Department of Food and Nutrition Science, College of Agricultural and Food Science, King Faisal University, Al-Ahsa, Saudi Arabia

**Keywords:** cardiometabolic health, chrononutrition, circadian misalignment, circadian rhythms, precision nutrition

## Abstract

Cardiometabolic diseases, including obesity, type 2 diabetes mellitus (T2DM), hypertension, and cardiovascular disease (CVD), remain major global health challenges despite widespread adoption of evidence-based dietary guidelines. Traditional nutrition recommendations have largely focused on dietary composition and energy intake, with limited consideration of the timing of food consumption. Growing evidence indicates that metabolic processes are strongly regulated by circadian rhythms, suggesting that when food is consumed may be a critical but underappreciated determinant of cardiometabolic health. Chrononutrition, which examines the interaction between meal timing and the circadian system, has therefore emerged as an important area of research. This narrative review synthesizes human evidence linking chrononutrition to cardiometabolic outcomes, with a focus on obesity, insulin resistance and T2DM, lipid metabolism, and cardiovascular risk. Findings from observational studies, randomized clinical trials, and mechanistic investigations consistently demonstrate that eating later in the biological day or night is associated with impaired postprandial glucose regulation, reduced insulin sensitivity, altered lipid handling, and adverse cardiometabolic profiles, independent of dietary composition and total energy intake. In contrast, eating patterns aligned with endogenous circadian rhythms characterized by earlier energy intake and avoidance of late-night eating appear metabolically favorable. This review further situates chrononutrition within the framework of precision nutrition. While precision nutrition aims to explain interindividual variability in metabolic responses using genetic, metabolic, and microbiome-based approaches, circadian timing is rarely considered. Because metabolic capacity varies across the day–night cycle, failure to account for meal timing, chronotype, and circadian alignment may contribute to unexplained variability in dietary responses. Integrating chrononutrition into precision nutrition frameworks may therefore improve interpretation of metabolic phenotypes and enhance the personalization of dietary strategies. Finally, key research gaps are identified, highlighting the need for long-term, diverse human studies and time-resolved metabolic phenotyping to clarify the role of chrononutrition in cardiometabolic disease prevention.

## 1 Introduction

Cardiometabolic diseases including CVD, T2DM, metabolic syndrome, hypertension, and obesity represent a growing global public health challenge (1). Collectively, these conditions are among the leading causes of morbidity and mortality worldwide and are closely interconnected, often sharing common underlying pathophysiological mechanisms such as insulin resistance, chronic low-grade inflammation, and endothelial dysfunction (2). Their development and progression reflect a complex interplay between non-modifiable and modifiable risk factors that shape both individual susceptibility and population-level disease burden (3). While non-modifiable factors such as age, sex, genetics, ethnicity, and family history are important for identifying high-risk individuals, modifiable lifestyle-related factors including diet, physical inactivity, poor sleep, chronic stress, and obesity remain central targets for prevention and intervention (4).

To mitigate the rising burden of cardiometabolic diseases, several evidence-based dietary patterns, including the Dietary Approaches to Stop Hypertension (DASH), Mediterranean, and plant-based diets, have been widely promoted (5, 6). Despite their proven benefits, global prevalence of obesity, T2DM, and CVD continues to increase (7), highlighting important limitations of conventional dietary guidelines. These guidelines have traditionally focused on dietary composition and energy intake while largely overlooking the timing of food consumption. Emerging evidence suggests that meal timing, eating frequency, and alignment with circadian rhythms represent critical yet underrecognized determinants of metabolic health. Circadian rhythms are natural, internal biological cycles that follow an approximately 24-h pattern and regulate key physiological processes, including sleep, hormone secretion, and metabolism. These rhythms persist in the absence of external cues but are synchronized by environmental signals, particularly the light–dark cycle. In humans, circadian rhythms are coordinated by a central clock located in the suprachiasmatic nucleus of the hypothalamus (8). Chronotype reflects an individual’s preferred timing of sleep and activity, typically described along a continuum from earlier to later circadian preference. Circadian misalignment arises when behavioral patterns, such as eating and sleeping, are not aligned with the internal biological clock (9). This temporal dimension of eating has become increasingly relevant in modern societies, which are characterized by irregular schedules, prolonged eating windows, and widespread circadian misalignment.

At the same time, the emergence of precision nutrition, which integrates genetic, metabolic, microbiome, and behavioral factors, offers new opportunities to better understand and target cardiometabolic risk (10). By tailoring dietary and lifestyle recommendations to an individual’s biological and environmental context, precision nutrition offers a more nuanced alternative to generalized dietary guidelines. However, while current precision nutrition frameworks increasingly incorporate omics-based markers and gut microbiota profiles (11), circadian biology remains relatively underexplored. Interindividual differences in circadian rhythms and chronotype (e.g., “early” versus “late” types) may substantially influence metabolic responses to food intake (12), yet these factors are rarely considered in personalized dietary recommendations.

This growing recognition has led to the emergence of chrononutrition, a field that examines how the timing of food intake interacts with circadian rhythms to influence metabolic health (13). Chrononutrition adds a temporal dimension to precision nutrition by emphasizing not only what and how much is eaten, but also when food is consumed in relation to the internal biological clock (14). Integrating chrononutrition principles into precision nutrition frameworks may therefore enhance the effectiveness of dietary strategies for the prevention and management of cardiometabolic diseases (15).

Accordingly, this narrative review synthesizes evidence from human observational and interventional studies examining chrononutrition, particularly the effects of eating during the biological day versus night, in relation to cardiometabolic health. The review focuses on obesity, insulin resistance, and cardiovascular disease risk, discusses underlying biological mechanisms, and highlights key research gaps and future directions for integrating meal timing into precision nutrition frameworks and cardiometabolic disease prevention strategies.

## 2 Literature search and study selection

This narrative review was conducted using a structured literature search to identify relevant human studies examining the relationship between meal timing, particularly eating during the biological day versus night, and cardiometabolic health. A narrative approach was selected to enable integration of evidence across heterogeneous study designs, including observational studies, randomized controlled trials, controlled feeding studies, and mechanistic investigations, which are central to chrononutrition research. In addition, variability in the definition and measurement of meal timing, circadian phase, and cardiometabolic outcomes across studies limits the feasibility of formal systematic synthesis. A narrative framework therefore allows critical interpretation and contextualization of findings, as well as integration of physiological mechanisms with clinical and epidemiological evidence.

Electronic databases, including PubMed/MEDLINE and Scopus, were searched up to December 2025. The search strategy was informed by a PICO framework, focusing on human populations (P), meal timing, chrononutrition, and circadian timing exposures (I), comparisons of early versus late or circadian-aligned versus misaligned eating patterns (C), and cardiometabolic outcomes (O). Search terms included combinations of “meal timing,” “time of eating,” “night eating,” “late eating,” “chrononutrition,” “circadian misalignment,” and “biological night,” along with outcomes such as “postprandial glucose,” “insulin sensitivity,” “lipid metabolism,” “obesity,” “blood pressure,” and “cardiometabolic risk”.

Animal-only and *in vitro* studies were excluded from the primary evidence synthesis but were considered where necessary to support mechanistic interpretation. Both primary studies and relevant evidence syntheses, including systematic reviews and meta-analyses, were considered where appropriate. Primary emphasis was placed on original human studies, particularly randomized controlled trials, controlled feeding studies, and well-designed observational studies.

Studies were selected based on relevance to circadian timing of food intake and cardiometabolic outcomes, including obesity, glucose metabolism, lipid profiles, blood pressure, and cardiovascular disease risk. Given the narrative nature of this review and the heterogeneity in study designs, exposure definitions, and outcome measures, a formal risk-of-bias assessment was not conducted. However, findings were interpreted in the context of study design, sample size, methodological rigor, and consistency across multiple lines of evidence, with greater weight given to controlled experimental studies.

## 3 Understanding chrononutrition and circadian regulation of metabolism

Chrononutrition is an emerging area of nutritional science that examines how the timing of food intake interacts with circadian rhythms to influence metabolic regulation (16). Human physiology is governed by an intrinsic near-24-h circadian system that coordinates biological processes including sleep–wake cycles, hormonal secretion, digestion, and energy metabolism (8). These rhythms are synchronized by environmental and behavioral cues, with light serving as the primary regulator of the central circadian clock and food intake acting as a major timing signal for metabolic tissues (17). Chrononutrition proposes that alignment between eating patterns and endogenous circadian rhythms may support metabolic efficiency, whereas habitual eating at biologically inappropriate times such as late at night or across irregular schedules may contribute to circadian misalignment and adverse cardiometabolic outcomes (18).

Metabolic regulation by the circadian system is hierarchically organized through a central clock located in the suprachiasmatic nucleus (SCN) of the hypothalamus, which coordinates peripheral clocks in key metabolic tissues including the liver, pancreas, adipose tissue, skeletal muscle, and intestine (19). These peripheral clocks regulate the rhythmic expression of genes involved in glucose and lipid metabolism, hormone secretion, appetite regulation, and energy expenditure (20). Because feeding timing strongly influences peripheral clock entrainment, food intake occurring at times misaligned with the light–dark cycle may disrupt coordination between central and peripheral circadian systems and impair metabolic regulation.

Importantly, peripheral clocks may also respond differently to feeding cues across tissues. For example, the liver clock is highly sensitive to meal timing and regulates rhythms in gluconeogenesis, glycogen storage, and lipid synthesis, while clocks in the pancreas, skeletal muscle, adipose tissue, and intestine influence insulin secretion, glucose uptake, substrate oxidation, adipokine release, and nutrient absorption. This tissue-specific entrainment may help explain why the same meal can produce different metabolic responses depending on circadian phase (21).

In human research, the concept of “biological night” can be operationalized using several approaches. Fixed clock-time definitions (e.g., late evening or night-time intake) are commonly used but may not account for interindividual differences in circadian phase. Chronotype-adjusted approaches define timing relative to individual sleep–wake patterns, such as eating in relation to sleep midpoint, providing greater personalization. More precise methods include physiological markers such as dim light melatonin onset (DLMO), which reflects internal circadian phase and provides greatest physiological precision but is less feasible in large-scale or free-living studies. Accordingly, most epidemiological and clinical studies rely on proxy measures that balance feasibility with biological relevance. In free-living and clinical settings, biological night is most commonly approximated using clock-time thresholds (e.g., food intake after 20:00–22:00) or timing relative to sleep (e.g., eating close to bedtime).

Circadian rhythms exert profound effects on metabolism through tightly regulated hormonal oscillations that align nutrient handling with the sleep–wake cycle. Key metabolic hormones, including insulin, glucagon, cortisol, leptin, ghrelin, and melatonin, exhibit robust daily rhythms that collectively regulate transitions between energy utilization and energy conservation across the day-night cycle. These coordinated hormonal signals ensure that glucose, lipid, and protein metabolism are optimized during the biological day and constrained during the biological night (22, 23).

During the morning and early active phase, metabolic regulation is characterized by a hormonal environment that promotes efficient nutrient utilization (24). Insulin secretion and peripheral insulin sensitivity are highest during this period, facilitating glucose uptake in skeletal muscle and adipose tissue, enhancing glycogen synthesis, and supporting anabolic metabolism. Concurrent suppression of glucagon limits hepatic glucose output when dietary glucose is available (20). The morning rise in cortisol further supports metabolic readiness by mobilizing energy substrates through increased gluconeogenesis and lipolysis, ensuring adequate fuel supply for daytime activity (25). Together, this hormonal alignment results in superior postprandial glucose tolerance, higher diet-induced thermogenesis, and more favorable lipid handling. Appetite-regulating hormones also reinforce daytime feeding behavior, with ghrelin levels rising prior to meals, particularly before breakfast to stimulate hunger, while leptin concentrations remain relatively lower, permitting energy intake during periods of high metabolic capacity (26).

As the day transitions into the biological night, the hormonal milieu shifts toward energy conservation and fasting physiology. Melatonin secretion increases in response to darkness, signaling the onset of the biological night to both central and peripheral clocks. Elevated melatonin suppresses insulin secretion and reduces insulin sensitivity, creating a metabolic state that is poorly suited for glucose handling (27). At the same time, glucagon levels increase, promoting hepatic glucose production to maintain normoglycemia during prolonged fasting (28). Leptin concentrations peak during the evening and overnight, suppressing appetite and supporting energy balance during sleep, while ghrelin levels decline, reducing hunger signals (29).

At the molecular level, metabolic hormones and nutrient signals interact with core clock genes, including CLOCK, BMAL1, PER, CRY, and REV-ERB, to regulate rhythmic expression of enzymes involved in glycolysis, gluconeogenesis, fatty acid oxidation, lipogenesis, and cholesterol metabolism (30, 31). Nutrient-derived signals such as glucose, amino acids, fatty acids, bile acids, and cellular energy sensors can also feed back on clock gene expression, reinforcing the bidirectional relationship between circadian timing and metabolism (32).

Collectively, these findings demonstrate that metabolic responses to food intake are influenced not only by dietary composition, but also by the timing of eating in relation to internal circadian biology. Eating at biologically inappropriate times may disrupt circadian coordination and impair metabolic regulation, highlighting the important role of meal timing in human metabolism.

## 4 Impact of circadian misalignment

Circadian misalignment refers to a mismatch between internal biological timing and external behavioral or environmental cues, such as eating, sleeping, or physical activity occurring at suboptimal circadian phases (33). This phenomenon is commonly observed in individuals engaged in shift work, those experiencing social jet lag, or individuals who habitually consume food late at night (34, 35). Eating during the biological night, when metabolic processes are less prepared to handle nutrient intake has been consistently associated with impaired glucose tolerance, reduced insulin sensitivity, and altered lipid metabolism (36). Over time, repeated circadian misalignment may contribute to the development of obesity, type 2 diabetes, hypertension, and cardiovascular disease (37).

Circadian misalignment also disrupts the rhythmic secretion of key metabolic hormones, including insulin, cortisol, melatonin, leptin, and ghrelin (33), thereby impairing coordination between the central circadian clock and peripheral clocks in metabolic tissues. This hormonal desynchronization disrupts coordinated metabolic regulation across central and peripheral tissues, impairing glucose handling, lipid metabolism, appetite control, and energy balance. As a result, nutrient intake occurs during periods of reduced metabolic capacity, leading to exaggerated postprandial glycemic and lipemic responses and diminished metabolic flexibility.

Evidence from controlled experimental models of circadian misalignment further supports these mechanistic pathways. Forced desynchrony, simulated night-shift, and late-dinner protocols have shown that eating at an adverse circadian phase can reduce glucose tolerance, impair insulin sensitivity, alter melatonin-insulin interactions, and exaggerate postprandial glycemic or lipemic responses even when energy intake and meal composition are controlled. Collectively, these findings suggest that meal timing can independently influence hormonal and metabolic responses under controlled dietary conditions (38).

**Figure 1:**
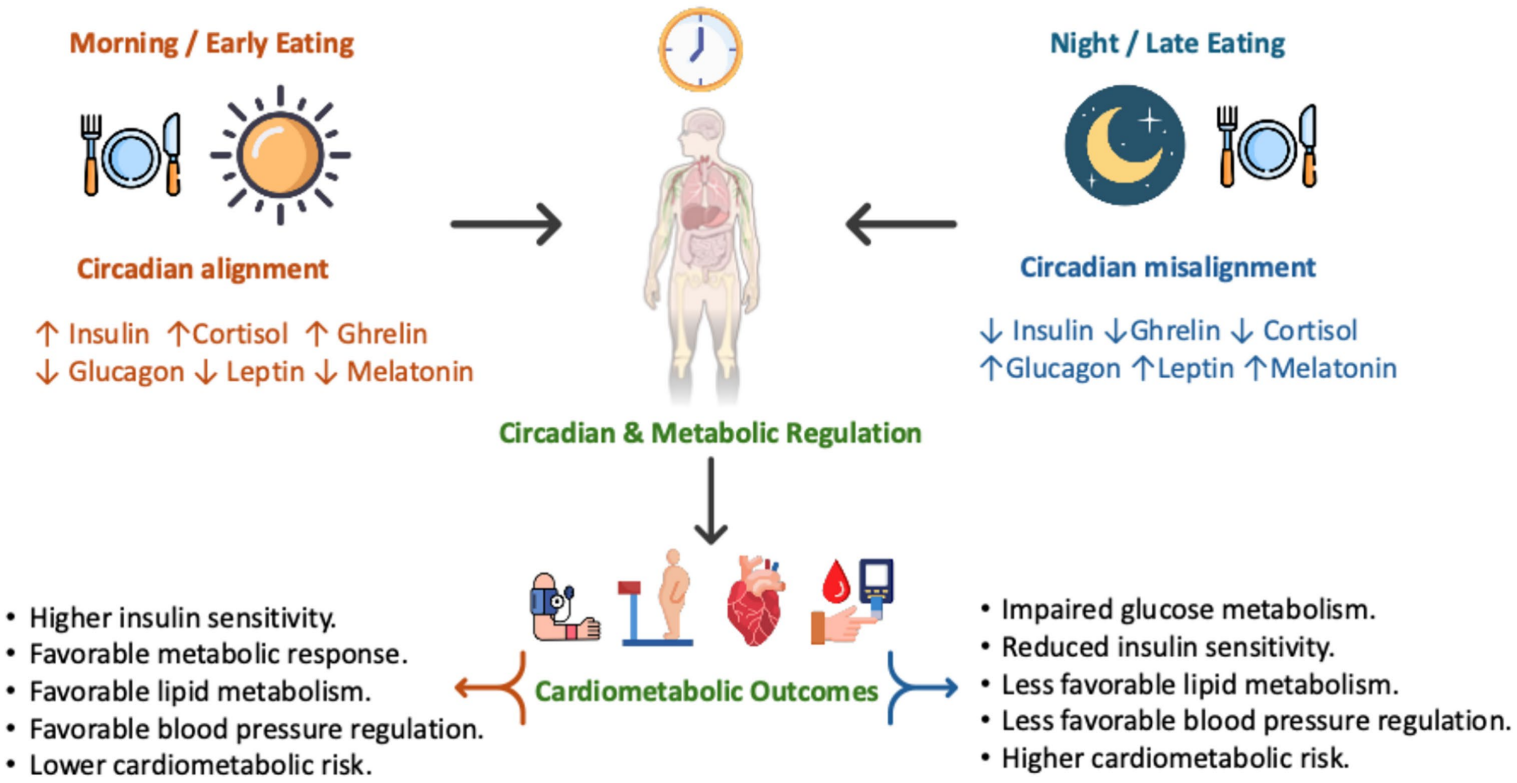
Conceptual framework linking chrononutrition, circadian regulation, and cardiometabolic health outcomes.

Beyond metabolic dysregulation, emerging evidence suggests that chronic circadian misalignment may promote low-grade systemic inflammation, oxidative stress, and alterations in gut microbiota composition (15), further amplifying cardiometabolic risk. These interconnected pathways provide biologically plausible mechanisms linking misaligned eating patterns to adverse cardiometabolic outcomes. The key processes through which circadian misalignment influences metabolic regulation and disease risk are summarized in Figure 1, which illustrates the interaction between meal timing, circadian clocks, and downstream cardiometabolic consequences.

Despite growing interest in this field, much of the existing human evidence is derived from observational studies and short-term experimental interventions. Nevertheless, findings from epidemiological, mechanistic, and controlled experimental studies collectively support the biological plausibility that circadian misalignment adversely affects metabolic regulation.

## 5 Chrononutrition and cardiometabolic health

### 5.1 Obesity

Evidence linking chrononutrition to obesity in humans is still developing, and randomized clinical trials remain scarce. Most available data come from observational and longitudinal studies, with relatively few directly comparing early versus late eating patterns in relation to body weight outcomes. Despite these limitations, a consistent pattern has begun to emerge, suggesting that delayed and irregular eating may be unfavorable for long-term weight regulation.

One observational study of 1,195 adults with overweight or obesity reported that later overall daily meal timing, assessed using the midpoint of meal intake, was associated with poorer long-term weight-loss maintenance over time. Each one-hour delay in the midpoint of daily food intake was linked to a 2.2% higher long-term body weight, and this relationship was particularly evident among individuals with a high genetic predisposition to obesity. In this subgroup, body mass index increased by more than 2 kg/m^2^ for every one-hour delay in meal timing, whereas no association was observed among those with lower genetic risk (39). These findings suggest that meal timing may interact with genetic susceptibility and may represent a modifiable behavioral factor relevant to personalized obesity management.

Additional evidence comes from studies examining night-eating behaviors. A recent scoping review found that night eating syndrome was positively associated with body mass index in most included studies, although results were less consistent in younger populations (40). This variability highlights the need for longer-term studies and experimental designs to better understand how night eating influences body weight across different age groups.

At the population level, similar associations have been observed. In a nationally representative analysis of 7,379 U. S. adults from NHANES 2015–2018, longer daily eating duration (>12 h), later timing of the last daily meal, and a later eating midpoint were associated with a higher prevalence of abdominal obesity and adverse metabolic outcomes. Individuals who consumed their last meal later in the evening or had a delayed midpoint of daily food intake were more likely to exhibit central adiposity, suggesting that extended eating windows and later overall food intake patterns may be unfavorable for body weight regulation (41). Consistent findings have been reported in other observational studies. For example, in a cohort of 872 adult women, greater energy intake earlier in the day and lower intake close to bedtime were associated with lower body mass index, whereas consuming a higher proportion of carbohydrates and protein near bedtime was linked to increased odds of overweight and obesity, particularly among individuals with a later chronotype (42). Similarly, a large longitudinal study of 8,153 adults reported higher obesity prevalence among individuals who regularly ate late dinners or consumed snacks after dinner (43).

Interventional studies examining energy distribution and meal timing provide further support for these observations. As summarized in Table 1, controlled feeding and randomized crossover studies suggest that concentrating energy intake earlier in the day may improve appetite regulation, metabolic efficiency, and body composition compared with patterns characterized by greater energy intake later in the day. Experimental evidence also indicates that late eating may reduce energy expenditure, alter appetite-regulating hormones, and impair substrate oxidation, potentially creating a physiological environment that favors positive energy balance and adiposity, even under isocaloric conditions.

**Table 1:** Summary of key human interventional studies examining the effects of meal timing on cardiometabolic outcomes.

Study	Design	*n*	Timing comparison	Outcomes	Key finding
Allison et al. (59)	RCT, crossover (8 wk)	12	Daytime (08:00–19:00) vs. Delayed (12:00–23:00)	Weight; insulin; lipids	Daytime → ↓ weight, ↓ insulin resistance, ↓ glucose; Delayed → ↑ TG
Bandín et al. (60)	RCT, crossover (2 wk)	32	Early lunch (13:00) vs. Late lunch (16:30)	Glucose; energy expenditure; substrate use; circadian markers	Late → ↑ glucose AUC; ↓ energy expenditure; ↓ CHO oxidation; altered cortisol
Centofanti et al. (48)	Controlled parallel trial (6 d)	55	Night fasting vs. Night snack vs. Night meal	Glucose; insulin; insulin sensitivity; NEFA	Night meal/snack → ↑ glucose AUC; ↑ NEFA; ↓ insulin sensitivity vs. night fasting
Črešnovar et al. (61)	Controlled parallel intervention (3 mo)	108	eTRE (8 h, early) vs. lTRE (8 h, late) vs. control (12 h ER)	Fasting glucose; BP; body composition	eTRE → ↓ glucose, ↓ fat mass, ↓ BP; no difference in weight vs. groups
Garaulet et al. (38)	RCT, crossover (Acute)	845	Early dinner (−4 h before sleep) vs. Late dinner (−1 h before sleep)	Glucose; insulin; *β*-cell function, melatonin	Late (↑ melatonin) → ↑ glucose AUC; ↓ insulin; stronger effect in MTNR1B carriers
Gu et al. (36)	RCT, crossover (Acute)	20	Routine dinner (18:00) vs. Late dinner (22:00)	Glucose; insulin; TG; FFA; cortisol; fat oxidation	Late → ↑ glucose; delayed TG; ↓ fat oxidation; ↑ cortisol
Hatamoto et al. (62)	RCT, crossover (8 d)	8	Early (08:30–19:30) vs. Late (12:00–23:00) schedule	24 h glucose	Late → ↑ mean 24-h glucose
Jakubowicz et al. (63)	RCT, parallel (12 wk)	60	Breakfast vs. Dinner kcal load	Glucose; insulin; insulin resistance; hormonal profile	Breakfast → ↓ glucose, ↓ insulin, ↓ androgens; ↑ SHBG
Jamshed et al. (64)	RCT, crossover (4 d)	11	eTRF (08:00–14:00) vs. Conventional (08:00–20:00)	24-h glucose (CGM); lipids; hormones; gene expression	eTRF → ↓ 24-h glucose; ↓ glycemic excursions; altered cortisol rhythm and circadian gene expression
Leung et al. (65)	RCT, crossover (4 wk)	19	Overnight fast (01:00–06:00) vs. usual night eating	Glucose; TG; weight	No change in glucose/TG; slight ↓ weight
Leung et al. (66)	Controlled crossover (acute)	10	Morning vs. Evening (± Midnight)	Glucose; insulin	Evening → ↑ glucose iAUC; ↑ insulin
Morgan et al. (67)	Controlled crossover (acute)	6	Early vs. Late energy distribution (high vs. low GI)	Glucose; insulin; TG; NEFA	Late → ↑ glucose; ↓ insulin sensitivity; effect amplified with high-GI meals
Morris et al. (68)	RCT, crossover (8 d)	13	Morning (08:00) vs. Evening (20:00)	DIT; metabolism	Evening → ↓ DIT (~50%); circadian-driven effect
Nakamura et al. (69)	RCT, crossover (Acute)	16	Early vs. Delayed dinner (up to 3 h)	Glucose (CGM); iAUC	Delay → ↑ peak glucose; ↑ iAUC
Sutton et al. (70)	RCT, crossover (5 wk)	8	eTRF (early 6 h window) vs. control (12 h)	Insulin sensitivity; β-cell function; BP; metabolic markers	eTRF → ↑ insulin sensitivity, ↑ β-cell function; ↓ BP
Vujović et al. (71)	RCT, crossover (Short-term)	16	Early vs. Late (isocaloric)	Appetite; energy expenditure; body temperature; gene expression	Late → ↑ hunger; ↓ energy expenditure; altered adipose gene expression

AUC, area under the curve; BP, blood pressure; CGM, continuous glucose monitoring; CHO, carbohydrate; DIT, diet-induced thermogenesis; eTRE, early time-restricted eating; FFA, free fatty acids; GI, glycemic index; HbA1c, glycated hemoglobin; iAUC, incremental area under the curve; lTRE, late time-restricted eating; NEFA, non-esterified fatty acids; RCT, randomized controlled trial; SHBG, sex hormone-binding globulin; TG, triglycerides.

Taken together, evidence from observational and controlled human studies suggests that late eating patterns, particularly delayed last meals, extended eating windows, and food intake close to bedtime, are consistently associated with higher obesity risk. Eating behaviors that align more closely with circadian rhythms, such as concentrating energy intake earlier in the day and avoiding late-night eating, appear to support healthier body weight regulation. However, because much of the available evidence remains observational and interventional studies are relatively short-term, causal relationships cannot yet be firmly established, underscoring the need for larger and longer-duration randomized trials.

### 5.2 Insulin resistance and diabetes

Among cardiometabolic outcomes, the relationship between chrononutrition, insulin resistance, and type 2 diabetes is supported by a relatively strong body of human evidence, including randomized clinical trials. One systematic review of randomized crossover studies comparing isocaloric, standardized carbohydrate-containing meals consumed in the morning versus the evening/night period in healthy, non-shift-working adults found a consistent pattern of poorer glycemic control with later intake. Across eight trials involving 116 participants, meals consumed during the evening/night period (primarily between 18:00 and 20:00 h) resulted in significantly higher postprandial glucose area under the curve compared with meals consumed in the morning (primarily between 07:00 and 11:00 h), with moderate certainty of evidence. In contrast, postprandial insulin responses did not differ significantly between morning and evening meals (44). These findings are consistent with impaired glucose handling during night-time carbohydrate intake despite relatively similar insulin responses.

Evidence from free-living and observational studies further supports the importance of circadian alignment of eating for glucose regulation. In a study of adults with overweight or obesity and diet- or metformin-controlled prediabetes or early type 2 diabetes (n = 26), habitual late eaters defined as consuming at least 45% of daily energy after 5 p.m. exhibited significantly higher postprandial glucose responses during an oral glucose tolerance test compared with earlier eaters. These differences were observed despite no differences in body weight, fat mass, total energy intake, or overall diet composition (45). Complementary findings were reported in a twin study of 92 adults, in which later circadian timing of the caloric midpoint relative to the individual’s internal clock was associated with lower insulin sensitivity, higher HOMA-IR, elevated fasting insulin concentrations, and greater body mass index and waist circumference. Notably, eating timing parameters showed moderate to high heritability and were closely linked to sleep timing, suggesting that genetic and circadian factors jointly influence vulnerability to insulin resistance (46). Collectively, these observational findings support associations between later eating timing, circadian phase, and impaired glucose regulation while also highlighting substantial interindividual variability in metabolic responses.

Acute postprandial studies provide further mechanistic support for these associations. A systematic review and meta-analysis examining identical test meals consumed during the day versus the night in healthy adults reported consistently poorer glycemic responses during night-time intake. Across 15 eligible studies, including 10 in the meta-analysis, postprandial glucose area under the curve was significantly lower during daytime consumption, indicating superior glucose tolerance earlier in the day. The meta-analysis also showed a modest but significant increase in postprandial insulin responses at night, although insulin findings were less consistent across individual studies (47). These postprandial findings provide mechanistic support for impaired glucose tolerance during biological night-time intake.

In addition to acute postprandial responses, interventional studies provide further evidence that circadian timing of food intake influences glucose metabolism beyond short-term meal responses. As summarized in Table 1, randomized crossover and controlled feeding studies consistently demonstrate that consuming meals earlier in the day or restricting food intake to earlier circadian phases improves glucose tolerance, insulin sensitivity, and 24-h glycemic control compared with later eating patterns. Importantly, several studies were conducted under standardized energy and macronutrient conditions, supporting the concept that meal timing itself independently contributes to metabolic regulation. Mechanistically, these effects have been linked to elevated melatonin levels during late eating, impaired insulin secretion, altered substrate utilization, and circadian misalignment. Nevertheless, findings remain somewhat heterogeneous across study designs, intervention durations, and study populations.

These controlled intervention findings are further reinforced by experimental models of circadian misalignment. In a controlled laboratory study involving 55 healthy adults exposed to simulated night-shift work, participants were randomized to fasting-at-night, snack-at-night, or meal-at-night conditions while maintaining energy balance and comparable macronutrient composition. Although night-shift work reduced insulin sensitivity across all conditions, glucose tolerance was significantly more impaired when food was consumed during the night compared with fasting at night. Importantly, fasting during night shifts attenuated postprandial glucose and lipid disturbances, highlighting night-time food intake as a key modifiable contributor to insulin resistance under conditions of circadian misalignment (48).

Evidence linking chrononutrition to the incidence of type 2 diabetes is more limited but is beginning to emerge from prospective studies. In the Whitehall II cohort of 2,642 adults with normoglycaemia at baseline, the timing of the last eating episode was not associated with overall risk of developing prediabetes or diabetes over 5 years. However, subgroup analyses revealed that women with lower baseline HbA₁c who consumed their last meal after 21:00 had a 51% higher risk of developing prediabetes or diabetes compared with those eating earlier in the evening (49). Additional population-based evidence comes from a cross-sectional analysis of 7,676 adults in the Henan Rural Cohort, where the dinner-bedtime interval was used as an indicator of circadian alignment. A longer interval between dinner and bedtime was associated with lower odds of type 2 diabetes, with individuals maintaining an interval longer than 3 hrs exhibiting approximately 23% reduced odds compared with those with shorter intervals (50). These findings suggest that eating closer to bedtime, and therefore deeper into the biological night, may be unfavorable for glucose metabolism and diabetes risk.

Overall, evidence from observational, mechanistic, and controlled human studies consistently indicates that glucose tolerance and insulin sensitivity are more favorable earlier in the biological day and progressively impaired later in the biological day and during the biological night. Although definitions of “late eating” and “biological night” vary across studies, findings from acute crossover trials, controlled feeding studies, and circadian misalignment models collectively support meal timing as an important determinant of glycemic regulation. These findings further suggest that recurrent food intake during adverse circadian phases may contribute to insulin resistance and long-term diabetes risk, particularly under conditions of chronic circadian misalignment.

### 5.3 Lipid profile and cardiovascular risk

Lipid metabolism is increasingly recognized as a circadian-regulated process, and emerging human evidence suggests that the timing of food intake influences postprandial triglyceride handling, substrate oxidation, and metabolic flexibility across the day-night cycle. Although direct evidence linking chrononutrition to hard cardiovascular disease outcomes remains limited, dysregulation of lipid metabolism and blood pressure represents biologically plausible pathways through which temporal eating patterns may contribute to long-term cardiovascular risk.

Mechanistic evidence from controlled human studies supports a clear time-of-day dependence in lipid handling. In a whole-room calorimetry study, Dörner et al. demonstrated pronounced diurnal variation in postprandial lipid metabolism under energy-balanced conditions. Eight healthy adults consumed three identical mixed meals evenly distributed across the day. Despite comparable caloric intake and insulin exposure, postprandial triglyceride metabolism varied substantially across the day and was accompanied by increased fat oxidation and a lower respiratory exchange ratio, indicating a progressive shift toward lipid utilization later in the day. Computational metabolic modeling further revealed enhanced triglyceride lipolysis, reduced exogenous triglyceride appearance from the gut, and increased endogenous lipid flux from the liver during evening hours (51). Together, these findings point to a circadian reorganization of lipid handling characterized by altered triglyceride turnover and impaired metabolic adaptation later in the day. Although derived from acute studies in healthy individuals, this work provides important insight into how repeated exposure to late-day or night-time eating could challenge lipid metabolic regulation over time.

Additional experimental evidence indicates that triglyceride metabolism is particularly sensitive to nocturnal food intake. In a tightly controlled constant-routine study involving 21 healthy adults, lipid profiles were assessed during habitual daytime eating and during nocturnal eating with hourly isocaloric meals. When participants consumed food at night, triglyceride concentrations reached levels comparable to daytime eating despite substantially lower caloric intake, suggesting an exaggerated triglyceride response during the biological night. Moreover, total 24-h triglyceride exposure was approximately 10% higher when meals were distributed across the full 24-h cycle compared with a conventional daytime eating pattern. Notably, endogenous circadian rhythms of triglycerides peaked at night and were phase-shifted by altered feeding schedules, whereas rhythms of total cholesterol, HDL-cholesterol, and LDL-cholesterol were relatively stable and peaked earlier in the day (52). These findings suggest that triglycerides, in particular, may represent a key lipid pathway linking night-time eating to dyslipidemia.

Prospective observational studies further support a potential link between temporal eating patterns and cardiovascular risk through established cardiometabolic intermediates. In the Health Professionals Follow-up Study, which followed 26,902 U. S. men free of cardiovascular disease at baseline for 16 years, breakfast skipping was associated with a 27% higher risk of incident coronary heart disease, while late-night eating was associated with a 55% higher risk compared with men who did not eat late at night. Importantly, these associations were attenuated after adjustment for body mass index, hypertension, hypercholesterolemia, and diabetes, suggesting that the relationship between meal timing and coronary heart disease risk may be mediated, at least in part, through established metabolic risk pathways rather than meal timing acting as an entirely independent risk factor (53).

Evidence linking chrononutrition to cardiovascular risk profiles, particularly blood pressure regulation, has also emerged from smaller prospective studies. In a one-year cohort study of 116 U. S. women (54), longer nightly fasting duration and later timing of the first eating occasion were consistently associated with poorer cardiovascular health scores and higher diastolic blood pressure in both cross-sectional and longitudinal analyses. Concentrating a greater proportion of daily energy intake in the largest evening meal was likewise associated with higher diastolic blood pressure over time, whereas higher eating frequency was inversely associated with blood pressure. Although circulating lipid concentrations were not directly assessed, the observed associations with central adiposity and blood pressure suggest overlapping metabolic pathways through which meal timing and prolonged nightly fasting may influence cardiovascular risk.

Emerging interventional evidence further supports a role for meal timing in lipid metabolism and cardiovascular risk regulation. As summarized in Table 1, controlled feeding and randomized crossover studies demonstrate that late eating may exaggerate postprandial triglyceride responses, delay lipid clearance, reduce fat oxidation, and alter cortisol rhythms, whereas earlier eating patterns and early time-restricted eating interventions have been associated with modest improvements in triglyceride handling, blood pressure regulation, and metabolic flexibility. Importantly, several studies reporting these effects were conducted under controlled or isocaloric conditions, suggesting that circadian timing may influence cardiometabolic risk independently of dietary composition alone. Nevertheless, findings remain heterogeneous and are often limited by short intervention durations and relatively small sample sizes.

Overall, evidence from mechanistic, observational, and controlled human studies indicates that lipid metabolism is strongly influenced by circadian timing, with triglyceride handling appearing particularly vulnerable to eating during the biological evening or night. Late or biologically misaligned eating appears to exaggerate postprandial triglyceride exposure and impair lipid turnover, even in healthy individuals under controlled conditions. While direct associations between chrononutrition and incident cardiovascular disease remain limited, consistent links with dyslipidemia, blood pressure, and central adiposity support the biological plausibility that temporal eating patterns contribute to cardiovascular risk.

## 6 Chrononutrition within precision nutrition

A central premise of precision nutrition is the recognition that individuals vary widely in their metabolic responses to food. However, most studies investigating this variability do not systematically consider when food is consumed or the internal circadian phase at which eating occurs. Postprandial responses are typically assessed using standardized test meals without accounting for time-of-day effects or individual circadian characteristics. As a result, daily variation in metabolic capacity driven by circadian oscillations in insulin sensitivity, hormonal secretion, and substrate metabolism remains poorly characterized. This omission may partly explain why individuals show heterogeneous postprandial glucose, lipid, appetite, and weight responses to similar dietary interventions even after accounting for diet composition, energy intake, genetics, microbiome profiles, and baseline metabolic phenotype. Accordingly, precision nutrition models may remain incomplete if they characterize what individuals eat without considering when eating occurs in relation to circadian phase.

Direct human evidence linking chrononutrition to gut microbiota rhythmicity is still limited, yet emerging observational studies point to meaningful time-of-day-dependent variation in microbial composition and function. In a controlled study by Kaczmarek et al. (55), fecal samples collected from 28 healthy adults at multiple time points revealed pronounced diurnal variation in both gut microbiota and microbial metabolites. Approximately one-third of bacterial operational taxonomic units varied significantly by time of day, while concentrations of key short-chain fatty acids including acetate, propionate, and butyrate declined progressively from morning to evening. Importantly, these microbial patterns were associated with eating behaviors, including eating frequency, earlier distribution of daily energy intake, and overnight fasting duration. Although the directionality and clinical significance of these associations remain unclear, the findings suggest that meal timing and eating regularity may influence microbial rhythmicity and metabolite production, potentially linking temporal eating patterns with host metabolic regulation. Experimental evidence further supports this concept. Thaiss et al. demonstrated that both mice and humans exhibit diurnal oscillations in gut microbiota composition and function, while circadian disruption induced by jet lag or altered feeding patterns impaired microbial rhythmicity and promoted metabolic disturbances, including glucose intolerance and increased adiposity (56). Together, these findings support a potential role of microbial rhythmicity as a mechanistic link between temporal eating patterns and metabolic regulation.

Genetic variation in circadian clock genes may further modulate chrononutrition-related behaviors and long-term metabolic outcomes, supporting the integration of circadian biology into precision nutrition frameworks. In the EPIC-Spain cohort (57), associations between variants in several circadian genes (including PER1, PER2, PER3, CRY1, NR1D1, and CLOCK), chronotype, eating behaviors, and obesity-related anthropometric outcomes were examined in over 3,000 adults. At nominal significance, a PER1 variant was associated with reduced long-term weight gain, while several CLOCK variants were linked to smaller increases in waist circumference over adulthood. Although these associations did not withstand correction for multiple testing, they highlight potential gene–behavior interactions between circadian genetics and meal timing, underscoring substantial interindividual variability in susceptibility to obesity in relation to chrononutrition patterns.

Emerging evidence suggests that gene-timing interactions may play an important role in modulating metabolic responses to meal timing. Variants in genes involved in circadian regulation and melatonin signaling, such as MTNR1B, have been associated with impaired glucose tolerance and increased risk of type 2 diabetes. Notably, melatonin signaling interacts with insulin secretion and sensitivity, and its effects may be particularly relevant during evening or night-time food intake when endogenous melatonin levels are elevated. Individuals carrying risk variants may be more susceptible to exaggerated glycemic responses when consuming meals late in the day. Future research should incorporate genetic stratification into chrononutrition study designs, for example through randomized crossover trials comparing early versus late meal timing across genotype groups. Such approaches may help clarify whether genetic variation modifies the metabolic effects of meal timing and support the development of more personalized chrononutrition strategies.

In addition to genetic influences on metabolic responses to meal timing, behavioral circadian traits such as chronotype further shape eating patterns and chrononutrition-related outcomes. In a cross-sectional study of 362 adults, individuals with a morning chronotype exhibited more regular meal patterns, higher breakfast frequency, lower night-eating syndrome scores, and less eating-related jetlag compared with evening chronotype individuals. Morning types also consumed a greater proportion of daily energy earlier in the day, whereas evening types concentrated energy intake later, particularly at dinner on weekdays (58). Importantly, aligning dietary guidance with chronotype should not be interpreted as encouraging late-night eating among evening chronotypes. Rather, chronotype-informed guidance may help tailor realistic strategies, such as gradual advancement of meal timing, improved breakfast regularity, reduced late-night intake, and better alignment between eating patterns, sleep timing, and circadian metabolic regulation.

Taken together, these human studies indicate that chrononutrition captures an important and underexplored source of interindividual variability in dietary responses. By integrating temporal eating patterns with genetic, metabolic, microbiome, and behavioral information, chrononutrition extends existing precision nutrition paradigms beyond nutrient composition alone. Although interventional evidence remains limited, incorporating circadian timing into personalized dietary strategies may enhance the ability of precision nutrition approaches to explain heterogeneous metabolic responses and to develop more effective, personalized, circadian-informed dietary interventions.

## 7 Current gaps and future directions

A key objective of precision nutrition is to explain interindividual variability in metabolic responses and cardiometabolic health outcomes. While major advances have been achieved through the integration of dietary composition, genetic variation, metabolomic profiles, and gut microbiota characteristics, a substantial proportion of variability in dietary responses remains unexplained. Chrononutrition represents a plausible but underrecognized contributor to this heterogeneity. Metabolic processes are strongly regulated by circadian rhythms, and accumulating evidence indicates that postprandial glucose and lipid metabolism, blood pressure regulation, and microbial metabolites vary according to meal timing and internal circadian phase. Yet, most precision nutrition studies do not account for these temporal factors, potentially misclassifying circadian effects as unexplained interindividual variability.

Despite growing interest, important gaps remain in the chrononutrition literature. Much of the current evidence is derived from cross-sectional analyses and short-term experimental studies, limiting causal inference and insight into long-term cardiometabolic outcomes. Many interventions are conducted under tightly controlled laboratory conditions in small samples of metabolically healthy individuals, which restricts generalizability to free-living populations and those at higher cardiometabolic risk. Longitudinal studies and randomized controlled trials with extended follow-up are needed to determine whether chrononutrition-based strategies lead to sustained improvements in obesity, insulin resistance, dyslipidemia, hypertension, and cardiovascular disease risk.

Another major limitation is the lack of diversity in study populations. Chrononutrition research has been conducted predominantly in Western, high-income settings, with limited representation of diverse ethnic groups, age ranges, socioeconomic backgrounds, and cultural eating practices. Because meal timing and daily routines are shaped by occupational demands, social norms, and cultural context, the applicability of current findings across populations remains uncertain. Addressing this gap will require culturally adapted study designs that better reflect real-world variability in eating behaviors and circadian misalignment.

An additional and underexplored gap relates to the limited characterization of diurnal variation in metabolic and hormonal responses to food intake. Many studies rely on single fasting measurements or aggregate daily outcomes, failing to capture within-day fluctuations in insulin sensitivity, lipid metabolism, vascular function, and key regulatory hormones. Consequently, the temporal dynamics of postprandial metabolism across the day-night cycle remain poorly understood, particularly in free-living populations. Future research incorporating time-resolved metabolic and hormonal assessments across different circadian phases is needed to clarify the biological mechanisms linking chrononutrition to cardiometabolic risk.

Progress in the field is also constrained by the lack of standardized and objective biomarkers to assess circadian eating behavior and circadian alignment. Reliance on self-reported meal timing introduces recall bias and measurement error, limiting precision and reproducibility. The development of objective biomarkers derived from time-resolved metabolomics, hormonal rhythms, or wearable technologies could substantially improve exposure assessment and advance chrononutrition research within precision nutrition frameworks.

To improve measurement precision in future chrononutrition studies, the integration of objective tools is essential. Continuous glucose monitoring (CGM) enables detailed assessment of glycemic responses across the day and in relation to meal timing. Actigraphy and wearable devices provide objective estimates of sleep–wake patterns and circadian alignment. Digital food logging, including smartphone-based applications with time-stamped entries, allows more accurate capture of meal timing in free-living conditions. Emerging passive sensing technologies may further enhance temporal resolution of dietary and behavioral data with reduced participant burden. In free-living research settings, a practical minimal measurement framework may involve combining at least one metabolic measure (e.g., CGM) with one behavioral measure (e.g., sleep and meal timing), balancing precision with feasibility in real-world settings.

From a translational perspective, effective implementation of chrononutrition will require flexible and culturally appropriate strategies. Optimal meal timing is unlikely to be identical across individuals and must accommodate diverse work schedules, social constraints, and cultural practices to ensure adherence and sustainability. Integrating chrononutrition principles into existing dietary guidelines, rather than proposing rigid or prescriptive timing rules, may enhance acceptability and real-world impact. Practical chrononutrition approaches, including time-restricted eating, early time-restricted eating, and avoidance of late-night food intake, represent translational strategies that directly target meal timing. Although a detailed evaluation of these interventions is beyond the scope of this narrative review, they provide important models for testing whether improving temporal alignment of food intake can translate into sustained cardiometabolic benefits.

Importantly, chrononutrition recommendations should also be tailored to individual circadian and clinical characteristics. For individuals with a late chronotype, gradual shifts toward earlier and more regular eating patterns may be more feasible than abrupt changes. In shift workers, complete circadian alignment may not always be achievable, and strategies may focus on minimizing food intake during the biological night while concentrating energy intake during periods of relative circadian alignment. In individuals using glucose-lowering therapies, including insulin or incretin-based medications, meal timing interventions should be approached cautiously to avoid adverse glycemic events, particularly when modifying evening or nocturnal eating patterns. These considerations highlight the need for flexible, individualized, and context-specific chrononutrition strategies.

In summary, advancing chrononutrition research will require longer-term, diverse, and methodologically robust human studies; improved characterization of diurnal metabolic and hormonal dynamics; development of objective biomarkers of circadian eating behavior; and translation into adaptable, real-world interventions. Incorporating chrononutrition into precision nutrition frameworks may be essential to more fully explain interindividual variability in cardiometabolic health and to enhance the effectiveness of personalized dietary strategies.

## 8 Conclusion

Chrononutrition adds an important temporal dimension to cardiometabolic health and precision nutrition. Current human evidence indicates that eating later in the day or during the biological night is associated with less favorable glucose regulation, insulin sensitivity, lipid handling, blood pressure regulation, and obesity-related outcomes, whereas earlier and more circadian-aligned eating patterns appear metabolically favorable. Although much of the evidence remains observational or short-term, mechanistic and controlled experimental studies support the biological plausibility that meal timing influences metabolic responses independently of diet composition and energy intake. Integrating circadian timing, chronotype, meal timing, and sleep–wake patterns into precision nutrition frameworks may improve interpretation of interindividual variability and support more personalized, feasible, and culturally adaptable dietary strategies for cardiometabolic disease prevention.
